# Differential mental health impact of cancer across racial/ethnic groups: findings from a population-based study in California

**DOI:** 10.1186/1471-2458-14-930

**Published:** 2014-09-08

**Authors:** Héctor E Alcalá

**Affiliations:** Department of Community Health Sciences, UCLA Fielding School of Public Health, Los Angeles, CA USA

**Keywords:** Cancer, Mental health, Stress process, Race/ethnicity

## Abstract

**Background:**

Little research has examined the interactive effect of cancer status and race/ethnicity on mental health. As such, the present study examined the mental health of adults, 18 and over, diagnosed with cancer. This study examined the extent to which a cancer diagnosis is related to poorer mental health because it erodes finances and the extent to which the mental health impact of cancer differs across racial/ethnic groups. Furthermore, this study aimed to test the stress process model, which posits that the proliferation of stress can lead to mental illness and this process can differ across racial/ethnic groups.

**Methods:**

Data from the 2005 Adult California Health Interview Survey was used (N = 42,879). The Kessler 6, a validated measure of psychological distress, was used to measure mental health, with higher scores suggesting poorer mental health. Scores on the Kessler 6 ranged from 0 to 24. Linear regression models estimating psychological distress tested each aim. The mediating effect of income and the race by cancer interaction were tested.

**Results:**

After controlling for gender, age, insurance status, education and race/ethnicity, cancer was associated with higher Kessler 6 scores. About 6% of this effect was mediated by household income (t = 4.547; SE = 0.011; p < 0.001). The mental health impact of cancer was significantly worse for Latinos and Blacks than for non-Hispanic Whites.

**Conclusions:**

The mental health impact of cancer is not uniform across groups. Future work should explore reasons for these disparities. Efforts to increase access to mental health services among minorities with cancer are needed.

## Background

With 50% of men and 33% of women in the US developing invasive cancer in their lifetimes [1], the mental health needs of this population deserves considerable attention. Available evidence suggests mental health problems are more prevalent among those who have been diagnosed with cancer than those who have not been diagnosed. Non-representative studies of cancer patients have shown rates of depression ranging from 15% to 25% [2], with higher rates among those with cancer recurrence [3]. Anxiety among cancer patients is more common than in the general population [4]. Increased mental health burden among cancer patients and survivors may be linked to increased stress from cancer, increased hopelessness when faced with death or increase in stress related to chronic pain [5].

Mental illness is problematic among those with cancer because it can pose new health problems or complicate existing ones. A diagnosis of depression has been linked to reduced quality of life, poorer compliance to treatment regimens, suicide and lengthened hospitalization [6]. Cancer patients suffering from major or minor depression have a 39% higher risk of dying that non-depressed cancer patients [7]. However, depression has not been consistently linked to progression of cancer [7]. Anxiety has been shown to impact several cancer related outcomes, with some effects frequently disappearing after accounting for depression [8].

Additionally, cancer can lead to a variety of adverse financial consequences. Cancer patients face hundreds of dollars in out of pocket expenses, even with publically funded health insurance [9]. To complicate things further, over a fourth of cancer patients lose their jobs within the first year of cancer diagnosis [10] and also face reduced pay even when remaining employed [11]. Finally, cancer can financially impact caregivers by requiring them to miss work in order to provide transportation for care [9]. This could further reduce the access to financial resources of the cancer patient.

### The role of race/ethnicity

The prevalence of mental illness varies considerably by race/ethnicity. In surveys of the general population several patterns emerge. African Americans and Latinos are less likely to suffer from depression and anxiety than non-Hispanic Whites (NHWs) [12, 13]. Asian Americans have lower rates of mental illness, when compared to Latinos [14]. The lower mental health burden among racial minorities is surprising given, minorities have less access to health care [15], lower socioeconomic status [16] and tend face higher levels of race-based discrimination [17], all of which are linked to poorer health. Some explanations for these observed differences include: culturally specific presentations of mental illness in minority groups [18], the protective effect of religious service attendance among African Americans [19], group differences in willingness to seek help in dealing with mental health issues [20, 21] and group differences in the perceived etiology and treatment of mental illness [22].

Even though racial/ethnic minorities suffer from lower levels of anxiety and depression than their non-Hispanic white counterparts, they still face many other challenges when it comes to access and utilization of mental health services. In terms of utilization, African American, Latino and Asian American teenagers with major depressive disorder have been shown to access mental health services at lower rates than their NHW counterparts [23]. Additionally, Latinos and African Americans are less likely to use specialty mental health care than their NHW peers, with differences not being completely accounted by insurance or morbidity [24]. This suggests minority groups may lack the appropriate level of care. Finally, minority groups may face barriers to care including limited English proficiency [25] and financial and non-financial barriers [26].

Despite extensive work in the field of cancer and mental health, little work has examined racial differences in mental health among those diagnosed with cancer. Among the available cross-sectional evidence, clear patterns do not emerge. Among low-income Latina and NHW women recovering from breast cancer, no differences in depressive symptoms are seen [27]. Conversely, among women receiving treatment for early stage breast cancer, African American women report significantly fewer depressive symptoms than both NHW and Latina women [28]. Also, among women with breast or gynecological cancer, no association between race/ethnicity and depressive disorders is observed [29]. Finally, higher levels if psychological distress are observed among African Americans with cancer when compared to NHWs with cancer [30].

Similarly, longitudinal studies of cancer patients have drawn contrasting conclusions. For example, elderly North Carolinians show comparable levels of depressive symptoms between African Americans and NHWs [31]. However, elderly colorectal cancer patients found that African Americans were more likely to suffer from depressive symptoms than their white counterparts [32].

The available studies examining racial differences in mental health among those diagnosed with cancer have many characteristics that do not permit assessment of racial ethnic differences at the population level. First, the vast majority of studies of those with cancer do not compare racial/ethnic groups [33, 34], focus on the comparison of only two groups [8, 27, 31, 35] or have small sample sizes that may prohibit accurate multi-group comparisons [29, 36]. Second, studies often focus on cancer of a specific site [28, 37], or among a certain age group [31, 38, 39], which can skew the prevalence of mental illness away from the population means. Third, much of the available work does not use samples that are representative of the population at large, thus limiting external validity. Finally, when differences are observed there is limited use of theory to explain differences.

### Theoretical framework and hypotheses

As a result of the gap in knowledge, this study aimed to explore the impact a cancer diagnosis has on the mental health of adults of different racial groups living in California. Specifically, this study examined the extent to which a cancer diagnosis is related to poorer mental health because it erodes finances, and the extent to which the mental health impact of cancer differs across racial/ethnic groups.

The overall theoretical framework guiding this study is based on the stress process model [40]. This model contends that primary stressors lead to secondary stressors via stress proliferation, which, lead to increased risk of negative mental health outcomes [40]. Stressors can be divided into two types: life events or chronic or repeated strains [40]. The former type of stressor has a fixed identifiable time point, while the latter occurs more subtly and is more persistent. Applied to the present study, the stress process model would view a cancer diagnosis as a primary stressor and life event.

Also key to the stress process model is the idea of secondary stressors. These stressors result from the primary stressor but may have a harsher impact than the initial stressor. In this study, a cancer diagnosis may lead to a loss of household income, which, in turn, may lead to additional negative events. While not under examination in the present study, the continued proliferation of stress can be buffered by psychological resources available to the individual and modified by ambient stressors [40].

Finally, the stress process model argues that all of these processes are impacted by social statuses, such as race/ethnicity, that lead to social stratification [40]. This may leave certain groups particularly vulnerable to the impact of either primary or secondary stressors. In the present study, racial groups facing persistent disadvantage may demonstrate an increased response to the primary stressor, increased stress proliferation or have access to fewer coping resources and thus have greater risk of poor mental health.

Based on the stress process model, three hypotheses were tested: 1) cancer diagnosis is associated with poorer mental health; 2) lower financial resources mediate this relationship; 3) there exists a race/ethnicity by cancer interaction, such that cancer has a more deleterious mental health impact for some groups. Given the relative social status of African Americans and Latinos, it is hypothesized that cancer has a greater impact on the mental health of these groups when compared to NHWs.

## Methods

### Data source

Publically available data from the 2005 Adult California Health Interview Survey (CHIS) was used in the present study. This cross-sectional telephone survey of California adults, age 18 and over, was conducted between July 2005 and April 2006. The CHIS was administered in English, Spanish, Mandarin, Cantonese, Vietnamese and Korean and was designed to be representative of California adults living in households [41]. The CHIS includes replicate weights to adjust for differential selection probabilities, non-response bias and stratification [41]. CHIS 2005 includes 80 replicate weights [42].

Overall, 43,020 adults completed the survey, and most missing data was imputed using hot deck imputation [41]. Mental health questions were not ascertained or imputed for respondents completing the CHIS via proxy, yielding missing data. For these analyses 141 proxy respondents were excluded, yielding an analytic sample of 42,879. Examination of excluded cases revealed older age and higher rates of cancer than included cases.

### Measures

**T**he Kessler 6 (K6), a psychological distress scale, was used to measure the primary outcome of interest. This validated scale is used to measure cases of diagnosable mental illness in population surveys [43]. Participants were asked six questions measuring nervousness, restlessness, hopelessness, sadness, worthlessness and feeling everything was an effort in the past 30 days. Each question ascertained the frequency on a 5-point Likert scale ranging from 0 to 4, with values of 0 indicating experiencing the dimension none of the time and values of 4 indicating experiencing the dimension all of the time. Scores on all questions were summed to create a 24-point scale (Cronbach’s alpha = 0.828). While this scale is traditionally transformed into a dichotomous variable at or above 13 to serve as a proxy for diagnosable mental illness [44], the continuous scale was retained. This increases statistical power and allows for the detection of subclinical differences in mental health that may still impact quality of life. Additionally, only 3.1% of the current study sample had a Kessler 6 score of 13 or greater, limiting the utility of this categorization in analyses. Non-dichotomous transformations of the K6 have been recommended and used [45, 46].

For these analyses the primary predictor, and thus primary stressor, of interest was having received a cancer diagnosis from a doctor. Participants were asked if they had ever been diagnosed with breast cancer. Additionally, they were asked if they had been diagnosed with any other cancer type and listed as many as six specific cancer types. While cancer is a very heterogeneous disease, sample sizes for individual cancer type were too small for analyses. Thus, responses were collapsed to create a dichotomous variable representing cancer and no cancer. Cases of non-melanoma skin cancer were coded as not having been diagnosed with cancer.

Annual household income in thousands of dollars was considered as a potential mediator and secondary stressor. This allowed for the measurement of proliferation of cancer related stress, via negative financial consequences that may occur. CHIS top coded all responses greater than 300,000 dollars per year as 300,000.

The main moderator and measure of social stratification of interest was race/ethnicity. Race/ethnicity was measured using a series of dummy variables representing the Office of Management and Budget’s race and ethnicity category combinations (i.e. NHW, non-Hispanic Black/African American, non-Hispanic Asian, non-Hispanic other race, non-Hispanic two or more races and Latino), where NHWs served as the referent category. These dummy variables were then multiplied with the cancer variable to create cancer by race/ethnicity interaction terms.

Control variables were included in the analyses after confirming they were significantly associated with psychological distress and cancer diagnosis. These included gender, current insurance status (insured versus uninsured), years of schooling, and age (18 to 29, 30 to 44, 45 to 59 and 60 and over). Years of schooling were originally coded into broad categories such that included: no formal education, 1–8 years, 9–11 years etc. However, these were recoded to represent values in the middle of the category to create a continuous variable. Reference groups used for categorical variables were: male, uninsured and age 18 to 29.

### Analyses

Analyses were conducted using Stata 12.1, using replicate weights. First, summary statistics were run for all variables. Then, a series of nested multiple linear regression models predicting psychological distress from cancer status were run. The first model included only cancer status as a predictor. The second model added gender, current insurance status, years of schooling, age and race/ethnicity as controls. The third model further added annual household income and tested for mediation using Sobel’s Test. The final model added interaction terms in order to test for moderation. Improvement in model fit was assessed by comparing R-square values and using adjusted Wald tests.

### Conceptual framework

**Figure 1:**
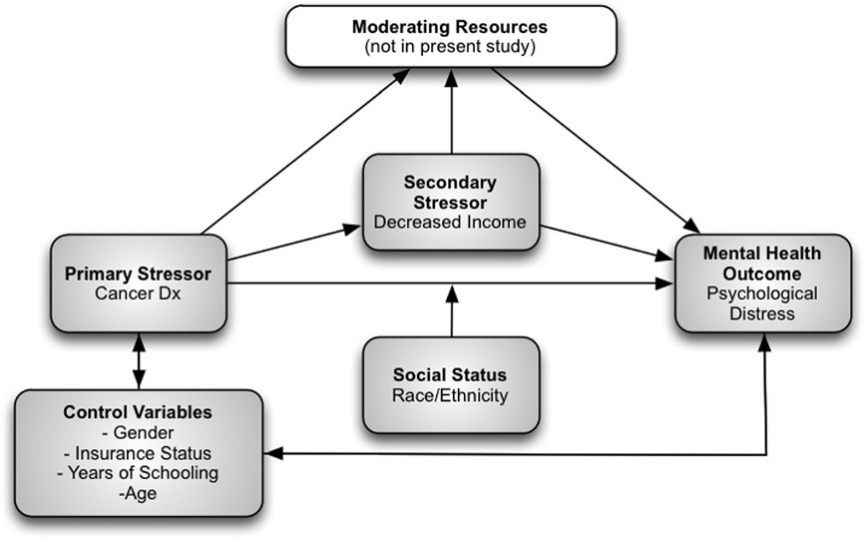
**Conceptual framework.**

Figure 1 depicts the conceptual framework for this study. Here cancer represents a primary stressor, which leads to decreased income as a secondary stressor. This secondary stressor then increases risk for psychological distress. Race, as social status, moderates the relationship between cancer and mental health. Control variables are shown to be associated with both the primary stressor and mental health. Finally, excluded from these analyses, but key in the stress process, is the idea that moderating resources can be tapped to attenuate the impact of stress and reduce the risk of mental illness via stress proliferation.

## Results

### Sample characteristics

As the weighted sample characteristics in Table 1 show, almost half of the sample was non-Hispanic White, less than a third was Latino and a sixth was Asian. All other racial categories comprised less than a tenth of the sample collectively. There were slightly more women than men. Approximately five sixths of the sample was currently insured. On average, participants had completed some college. Less than one third of respondents were between 30 and 44 years of age, one fourth was between 45 and 59, one fifth was 60 and older. The prevalence of cancer was low in the sample, with less than seven percent of adults reporting lifetime diagnosis. The average K6 score was below 3.5, indicating low levels of psychological distress.

**Table 1:** **Summary statistics for CHIS 2005 analytical sample**

		Unweighted			Weighted	
Characteristic	Level	N	Proportion/Mean	Median (Range)	Proportion/Mean	Robust SE
Race	White	27,511	0.642	-	0.487	0.000
	Latino	8,020	0.187	-	0.309	0.000
	African American	1,813	0.042	-	0.056	0.000
	Asian	3,875	0.090	-	0.125	0.000
	Multiracial	1,230	0.029	-	0.014	0.000
	Other	430	0.010	-	0.010	0.000
Gender	Male	17,394	0.406	-	0.490	0.000
	Female	25,485	0.594	-	0.510	0.000
Current insurance status	Insured	38,056	0.888	-	0.838	0.003
	Uninsured	4,823	0.113	-	0.162	0.003
Years of education		42,879	13.801	13 (20)	13.104	0.015
Household income^a^		42,879	68.810	50 (300)	66.936	0.393
Age	18-29	5,142	0.120	-	0.229	0.000
	30-44	11,307	0.264	-	0.316	0.001
	45-59	13,298	0.310	-	0.256	0.001
	60+	13,132	0.306	-	0.198	0.001
Kessler 6		42,879	3.293	2(24)	3.383	0.026
Cancer	Yes	4,229	0.099	-	0.066	0.001
	No	38,650	0.901	-	0.934	0.001

Note: Totals may not sum to 1 due to rounding.

^a^In thousands of dollars.

### Cancer and mental health

Table 2 shows the four different regression models predicting K6 score. Overall, each successive model was an improvement over the last, both in terms of variability explained and added variables being significant improvements. Model 1 shows a very small association between cancer and K6 score, such that having cancer was associated with four tenths of a unit increase on the scale (b = 0.395; p < 0.01). Model 2 shows, after controlling for age, gender, years of education and race/ethnicity, the association between cancer status and K6 score increases by 100%, such that having cancer is associated with eight tenths of a unit increase on the scale (b = 0.792; p < 0.001). Female gender, being currently insured, African American race, Multiracial identification and identifying with “Other” race were associated with higher K6 scores. Latino ethnicity and being in an age group over 30 were associated with lower scores.

**Table 2:** **Nested regression models predicting Kessler 6 score from cancer status, CHIS 2005 (N = 42,879)**

		Model 1			Model 2			Model 3			Model 4		
Independent Variable^a^	Level	b	Robust SE	p	b	Robust SE	p	b	Robust SE	p	b	Robust SE	p
Cancer	Yes	0.395	0.113	**	0.792	0.117	***	0.744	0.115	***	0.382	0.106	**
Gender	Female				0.507	0.056	***	0.427	0.055	***	0.425	0.055	***
Age	30-44				−0.337	0.084	***	−0.239	0.083	**	−0.243	0.083	**
	45-59				−0.265	0.085	**	−0.132	0.086		−0.140	0.086	
	60 and over				−1.202	0.102	***	−1.332	0.104	***	−1.325	0.103	***
Years of education					−0.123	0.011	***	−0.077	0.011	***	−0.076	0.010	***
Currently insured	Yes				−0.357	0.093	***	−0.162	0.095		−0.175	0.096	
Race	Black				0.565	0.152	***	0.351	0.148	*	0.251	0.151	
	Latino				−0.267	0.081	**	−0.436	0.083	***	−0.509	0.086	***
	Asian				0.083	0.088		−0.028	0.091		−0.061	0.093	
	Multiracial				0.734	0.172	***	0.649	0.172	***	0.632	0.176	**
	Other				0.913	0.299	**	0.805	0.294	**	0.636	0.294	*
Household income^b^								−0.009	0.000	***	−0.009	0.000	***
Race*Cancer	Black*Cancer										1.484	0.590	*
	Latino*Cancer										1.460	0.365	***
	Asian*Cancer										0.263	0.402	
	Multiracial*Cancer										0.031	0.525	
	Other *Cancer										2.435	1.989	
Constant		3.357	0.027	***	4.909	0.194	***	4.916	0.192	***	4.959	0.192	***
Model Statistics													
F		12.260		***	36.720		***	62.730		***	44.660		***
df		1,79			12, 68			13, 67			18, 62		
R^2^		0.001			0.032			0.047			0.048		
Model Comparison^c^													
F					37.190		***	338.220		***	2.540		*
df					10, 70			1, 79			4, 76		

Note: b = beta coefficient; SE = standard error; AA = African American; * ≤ .05. ** ≤ .01.*** ≤ .001.

^a^Reference categories: cancer = no; age = 18-29; currently insured = no; race = non-Hispanic White; Cancer*Race = no cancer*non-Hispanic White.

^b^In thousands of dollars.

^c^Model 2 compared to Model 1; Model 3 compared to Model 2; Model 4 compared to Model 3.

### Decreased household income as a mediator

Model 3 tests for the mediating effect of household income. In this model, insurance status was no longer significantly associated with the outcome. Cancer was associated with three fourths of a point increase in Kessler 6 score (b = 0.744; p < 0.001) and a 1,000 dollar increase in household income was associated with a small decrease in Kessler 6 score (b = −0.009; p < 0.001). Sobel’s Test revealed that income was a significant mediator (t = 4.547; SE = 0.011; p < 0.001). Approximately 6.061% of the effect of cancer on K6 score was mediated by income [((0.744 - 0.792)/ 0.792)*100)], suggesting most of the effect of cancer on Kessler 6 score occurs directly, or is mediated by other factors.

### Race/ethnicity as a moderator

**Figure 2:**
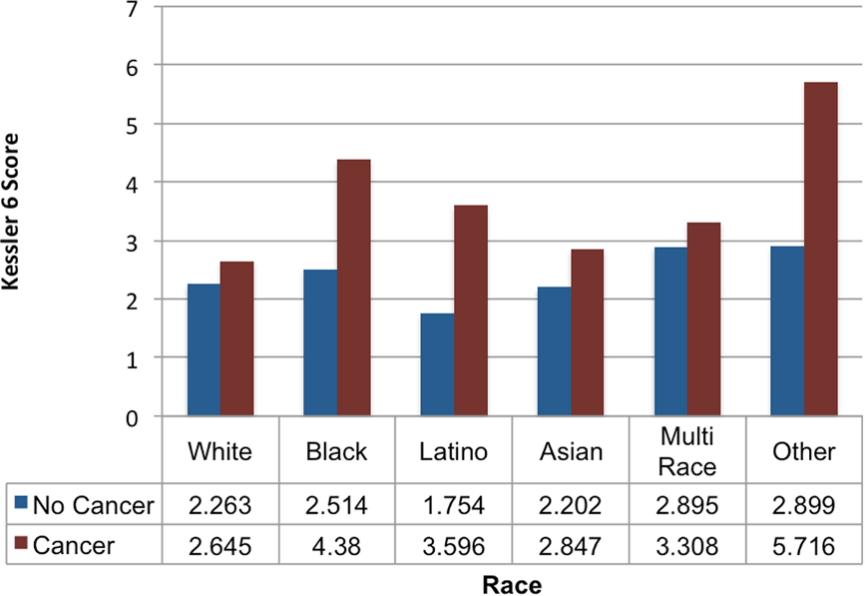
**Estimated Kessler 6 (K6) score by race/ethnicity and cancer status.**

In Model 4 in Table 2, lifetime cancer diagnosis was associated with significantly higher Kessler 6 scores among NHWs. Post-hoc analyses (not shown) showed that a lifetime cancer diagnosis status was associated with higher K6 scores among African Americans and Latinos. Individual interaction terms reveal that Latinos and African Americans both see a greater increase in K6 score when diagnosed with cancer than NHWs. Post hoc analyses (not shown) revealed that Latinos see a greater increase in K6 scores when compared to individuals with multiracial identification and Asians. Finally, those in the “Other” racial category saw the greatest, but not significant, impact of cancer diagnosis. Figure 2, depicts the predicted K6 score across cancer status and racial/ethnic categories for women, 60 and over, with 16 years of education, insurance and 45,000 dollars of household income. Here NHWs have similar K6 scores with and without cancer. Conversely, African Americans and Latinos, see much higher K6 scores when they have cancer.

## Discussion

Results demonstrated cancer was associated with higher levels of psychological distress. However, this was not true in all racial/ethnic groups, suggesting group differences in response to cancer. Higher levels of distress were found for NHWs, African Americans and Latinos with cancer, when compared to their cancer free peers.

Mediation analyses provided support for the predictions made by the stress process model. Findings suggest that cancer diagnosis was associated with worsened mental health, because of a reduced income. Thus, the idea that a cancer diagnosis leads to decreased household earnings is supported. This may occur for a variety of reasons including decreased ability to earn money or increased expenses [9, 11]. While these specific mechanisms were not tested, findings highlight the importance of adequate financial support to those dealing with cancer. Additionally, because findings reflect the impact of lifetime cancer diagnosis, financial ramifications of cancer may be long lasting.

Moderation analysis revealed differential mental health impact of cancer across racial/ethnic groups, yielding additional support for the stress process model. African Americans and Latinos exhibited more detrimental mental health impact of cancer, when compared to non-Hispanic Whites. This is consistent with the stress process model, where groups with lower social standing differentially experience primary stressors, stress proliferation and the development of mental illness. That is, certain racial ethnic groups may possess differential exposure to and susceptibility to stress.

Because the present study did not analyze reasons for observed disparities, cautious interpretations are recommended. Attributing observed differences to cultural differences is problematic because racial/ethnic groups presented are not homogeneous. One cannot discount the possibility that observed differences may be due to differential discrimination in the medical setting, different stage of cancer diagnosis or differing quality of care. Future work should attempt to determine reasons for the observed differences. Additionally, given the large, but non-significant, effect of cancer diagnosis seen among those in the “Other” racial/ethnic group, future studies should attempt to disaggregate this group with larger sample sizes.

Despite the important findings of this study, it has several limitations. Several stem from the fact that the data is cross-sectional in nature. As such, it is impossible determine causal relationships from the data or establish temporal sequence of events. Particularly, it is possible the proposed linkage between cancer and mental health works in reverse. That is, poor mental health, leads to diminished resources, which leads to increased risk of developing cancer. However, use of the Kessler 6, which limits its assessment of mental health to the past month, should help limit the impact of reverse causality, since the majority of cancer diagnoses are unlikely to have occurred in the past month. Furthermore, the cross-sectional nature of the survey also means measures may suffer from a differential degree of recall bias. Unfortunately, there is no way to ascertain this impact from the present study, and thus suggests future work should focus on prospective study designs. Finally, because the study uses a lifetime cancer diagnosis, it is impossible to know if there is a key timeframe in which cancer impacts mental health.

The sampling frame used in the CHIS and missing data is likely to produce an underestimate of the relationship between cancer status and mental health. Those with advanced cancers and those with serious mental illness are likely excluded from the frame or analytic sample because they may be living in institutions, may suffer illness so severe that it prevents them from responding to telephone surveys, they do not live in a household or had missing mental health data. As such, findings likely represent an underestimate of the actual relationship between cancer and mental health.

Finally, key measures in this study also have important limitations. The K6 scale, provides a general measure of mental health that does not allow for the measurement of specific mental illnesses. Also, use of a continuous scale departs from the standard paradigm emphasizing clinical level changes in mental health. However, when Model 4 was repeated using the standard dichotomous version of the Kessler 6 (i.e. scores of 13 or greater denoting serious psychological distress), the effect of cancer was still greater for African Americans and Latinos when compared to NHWs, suggesting clinical and subclinical effects occur in a similar pattern. The measurement of cancer is limited because it does not indicate current cancer status, cancer treatments that have been received, time since diagnosis, stage and cancer type. While the type was deliberately ignored in these analyses to bolster sample size, it is likely that this and the other aforementioned characteristics of a person’s cancer can moderate the relationship between cancer and mental health. Additionally, time since diagnosis, stage and treatment can provide valuable information, but this information was only available for women reporting breast cancer. Finally, the use of racial categories in this study should be interpreted with caution because the broad racial categories utilized encompass a vast amount of within group heterogeneity.

## Conclusions

Overall, this study suggests that African Americans and Latinos are more vulnerable to adverse mental health impacts due to cancer, when compared to non-Hispanic Whites. As such, efforts to bolster financial and psychosocial coping resources among these groups should be explored. This is especially important given the lower odds that minorities with advanced cancer have of receiving mental health services when compared to non-Hispanic Whites with advanced cancer [36]. As such, access mental health services in these groups should be promoted.
